# Site‐Specific Hydroxide Formation and Corrosion on Mg Nanocrystals

**DOI:** 10.1002/advs.75169

**Published:** 2026-04-27

**Authors:** Yao Liu, Zongmin Sun, Chenhao Wu, Kun Yang, Zhiyuan Ge, Chenyu Wang, Wenpei Gao, Jianbo Wu, Xiaoqin Zeng

**Affiliations:** ^1^ National Engineering Research Center of Light Alloy Net Forming School of Materials Science and Engineering Shanghai Jiao Tong University Shanghai P. R. China; ^2^ Shanghai Key Laboratory of Hydrogen Science & Center of Hydrogen Science State Key Laboratory of Metal Matrix Composites Shanghai Jiao Tong University Shanghai P. R. China; ^3^ State Key Laboratory of Metal Matrix Composites Future Material Innovation Center Zhangjiang Institute for Advanced Study Shanghai Jiao Tong University Shanghai P. R. China

**Keywords:** 3D tomography, corrosion, interfacial reactions, magnesium, quasi in situ

## Abstract

Magnesium is one of the lightest structural metals and is highly attractive for aerospace, transportation, biomedical, and energy technologies, yet its application is limited by rapid corrosion and an incompletely understood initiation mechanism. Here, using single‐crystalline Mg nanocrystals as a defect‐free model system, we identify intrinsic corrosion behavior. Results show that the alkali‐induced Mg(OH)_2_ hydration layer initially functions as a protective barrier, but its stability is determined by interfacial coherence with the Mg substrate. Once the film grows beyond a critical thickness of about 10–20 nm, incomplete coverage at crystal corners leads to local exposure. Quasi in situ TEM and 3D reconstruction reveal that corrosion starts from these exposed corners, propagates along junctions between the thick hydroxide and Mg underneath, and generates interfacial rupture that acts as pathways for Cl^−^ ingress. Rather than dissolving the entire hydroxide film, Cl^−^ ions preferentially attack incoherent Mg(OH)_2_/Mg interfaces, triggering localized matrix corrosion. These findings provide direct experimental insight into the earliest stages of Mg corrosion and identify interfacial engineering as a viable strategy to enhance corrosion resistance in Mg and potentially other lightweight metals.

## Graphical abstract

Using single‐crystalline Mg nanocrystals, this study uncovers the intrinsic corrosion initiation mechanism. The stability of the protective Mg(OH)_2_ film depends on its interfacial coherence with the Mg substrate. Corrosion initiates at exposed corners, where film coverage is incomplete. Chloride ions then exploit ruptures at incoherent Mg(OH)_2_/Mg interfaces to trigger localized corrosion, indicating that interfacial engineering is a key resistance strategy.

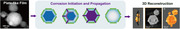

## 1 Introduction

Lightweight structural materials are essential for sustainable development, particularly in transportation, aerospace, and energy sectors, where reducing weight directly improves fuel efficiency and reduces emissions. Among them, magnesium and its alloys are highly attractive owing to their low density and high specific strength [1, 2]. However, their broader implementation is hindered by rapid and unpredictable corrosion, especially in chloride‐rich environments such as marine and physiological conditions [3, 4, 5, 6, 7]. Although thermodynamics can calculate and predict the stability window of the passivating Mg(OH)_2_ hydroxide film in alkaline conditions, it does not explain how the film forms, evolves, and breaks down when exposed to chloride [8, 9, 10, 11]. Similarly, classical theory based on the Pilling‐Bedworth ratio (PBR) suggests that the Mg(OH)_2_ film (∼1.77) should be compact and protective, unlike the porous MgO layer (∼0.81) [12, 13]. However, this macroscopic theoretical stability contradicts the limited protection observed in practice. Because corrosion starts at the surfaces and interfaces, the nanoscale processes, including hydration, hydroxide nucleation, and ion penetration, ultimately determine the macroscopic corrosion morphology and kinetics. However, the initial stage remains poorly understood because available studies using polycrystalline materials couple the influences of crystallographic orientation, defects, and nanoscale curvature, making it difficult to isolate intrinsic dissolution behavior, hydroxide growth modes, and Cl^−^ invasion pathways in Mg [14, 15, 16]. Macroscopic electrochemical measurements give contradictory reports; for instance, the basal (0001) plane was identified as both the most and the least corrosion‐resistant [17, 18, 19, 20]. In addition, the sample preparation using focused ion beam (FIB) can often create non‐native interfaces and damaged layers, which complicates the interpretation of interfacial structure and chemistry. As a result, the microstructure of the nascent hydroxide film, its formation mechanism, and the corresponding structure‐property relationships remain insufficiently resolved, leaving a critical gap in understanding the Mg corrosion [21, 22, 23].

To decouple these intertwined factors requires model systems with atomically defined surfaces. Single‐crystalline Mg nanocrystals provide an ideal platform for this purpose, particularly given their hexagonal close‐packed (hcp) structure and facet‐dependent passivation behavior. The single crystalline Mg also enables the corrosion behavior to be isolated from microstructural heterogeneities such as grain boundaries, dislocations, and surface roughness. Its well‐defined morphology and surface facets allow the size effects and site‐specific behavior to be systematically studied [24, 25, 26]. The absence of grain boundaries permits direct observation of orientation‐dependent hydroxide formation and dissolution, making crystallographic orientation an independent and controllable variable [27, 28, 29]. The nanoscale dimensions allow for high‐resolution imaging using advanced transmission electron microscopy (TEM) [30] without FIB thinning, thus preserving native interfaces and hydration layers. Collectively, these strengths make the single‐crystalline Mg nanoparticle ideal to quantitatively correlate the structure factors, including surface facets and film integrity, with corrosion behavior at the mesoscale.

Herein, we investigate the formation and chloride‐induced degradation of interfacial hydroxide films on specific crystallographic facets of single‐crystalline magnesium nanocrystals. By combining structural characterization and quasi‐in situ corrosion experiments, we quantify the influence of microstructure and orientation of Mg on the passive film formation, and the effect of film thickness on corrosion. 3D reconstruction from TEM imaging allows us to track the corrosion propagation along the original atomic interfaces and correlate it with local film stability. Results show that the interface coherence and stress determine the morphology and integrity of the hydroxide film, leading to site‐specific protection against corrosion. When the passive film exceeds a critical thickness, its bending rigidity and the resultant tensile stress from substrate undulation induce nanoscale spalling. Chloride ions can subsequently infiltrate and react with Mg along the Mg(OH)_2_/Mg interface, followed by localized metal corrosion. Therefore, through integrated microscopy analysis of corrosion in single‐crystalline Mg nanocrystals, we reveal how interfacial coherence governs the failure of passive films and propose it as a key design parameter for corrosion‐resistant magnesium and other lightweight metals.

## 2 Results and Discussion

### 2.1 Facet‐Dependent Growth of Hydroxide Passivation Layers on Mg Nanoparticles

**FIGURE 1:**
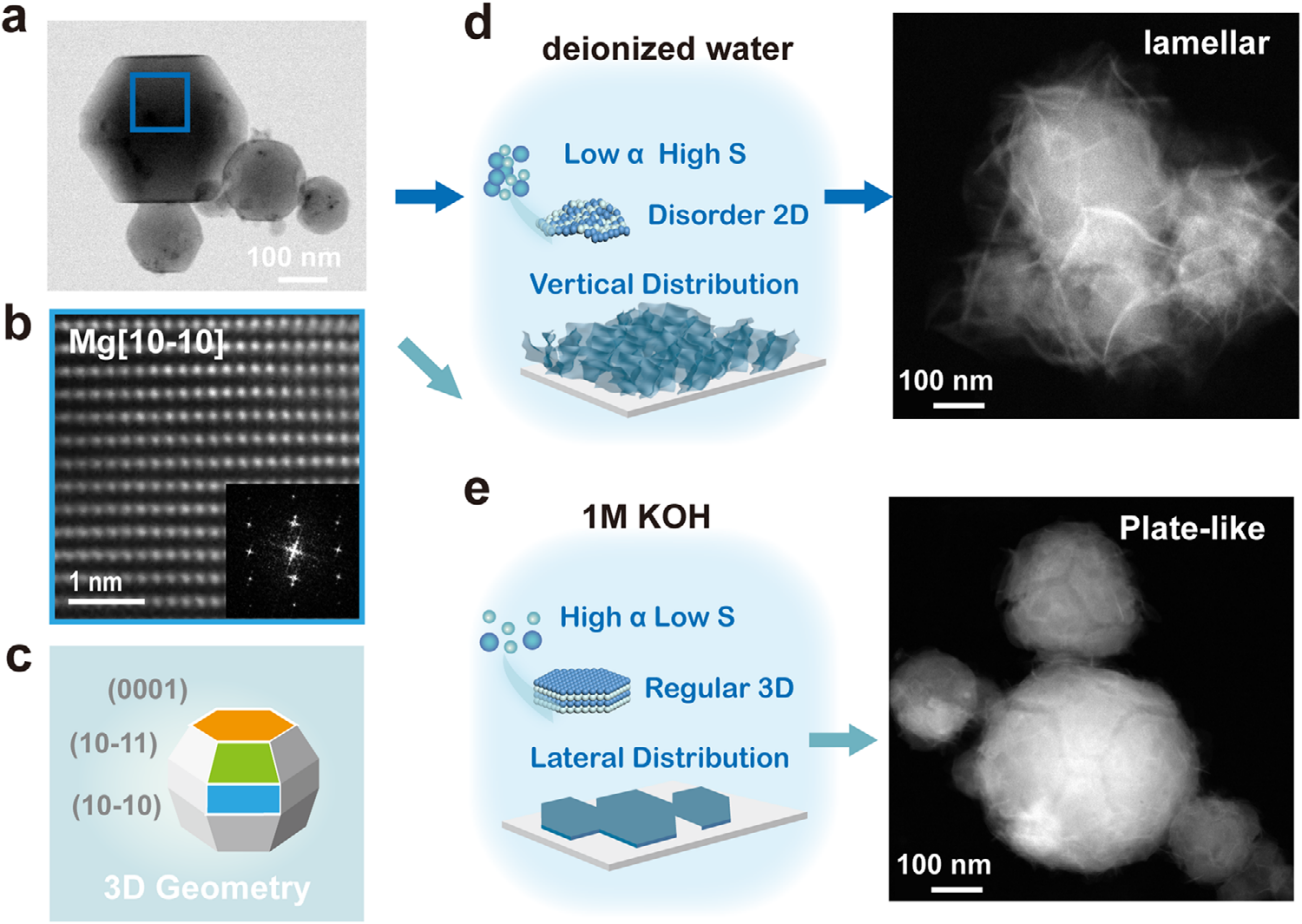
(a) TEM and (b) HRTEM images with the corresponding FFT pattern of Mg nanoparticles. (c) 3D geometric model illustrating the crystallographic orientation of the nanoparticles. HAADF‐STEM images and associated schematics of Mg nanoparticles after 5 min of immersion in (d) deionized water and (e) 1 m KOH, demonstrating the control of film morphology through supersaturation (S) and electrolytic dissociation (α).

Single‐crystalline Mg nanoparticles were synthesized using a direct current (dc) arc plasma method [31]. As illustrated in Figure 1a and Figure S1, the particles present a 3D faceted morphology with 6‐fold symmetry and sizes of hundreds of nanometers in diameter. For the nanoparticle enclosed by the blue frame, its crystallographic characteristics were further analyzed via high‐resolution transmission electron microscopy (HRTEM), as shown in Figure 1b. Figure 1c depicts the corresponding crystallographic structure, where the three characteristic crystal planes {0001}, {10‐10}, and {10‐11} are labeled in orange, blue, and green, respectively. To investigate the film formation characteristics at the initial corrosion stage, we study the morphology and analyze the corrosion products formed on the specimens 5 min after immersion in salt‐free aqueous environments. In deionized water, the nanoparticles are covered by 2D wrinkled lamellar films through anisotropic growth, which completely encapsulates the particles. In comparison, in alkaline (1 m KOH), 3D plate‐like structures form. The newly formed structures are site selective; they form continuous layers on planar facets, while distinct nanoscale spalled areas are present at edges and corners. Additional SEM images of these spalled areas at the nanoscale are shown in Figure S2.

The different growth behaviors can be comprehended using the dual‐parameter framework of electrolytic dissociation (α) and supersaturation (S), as shown in Figure 1d,e. The 2D wrinkled lamellar film morphology in deionized water originates from the synergistic effects of a weak electrolytic environment (low α) and high supersaturation (high S) conditions. The rapid corrosion kinetics in deionized water generate substantial supersaturation, which drives fast and disordered anisotropic growth of hydroxide along preferential crystal planes. This observation is consistent with reported low α/high S regimes that favor the formation of kinetically controlled 1D/2D structures [32]. In contrast, the growth dynamics of corrosion products in 1 m KOH solution undergo a transformation, which is driven by the combined effects of strong electrolytic dissociation (high α, α≈1) and passivation‐induced reduction in supersaturation (low S). The high OH^−^ concentration in the alkaline solution facilitates complete ionic dissociation, a process that promotes isotropic 3D nucleation. Meanwhile, the formation of a protective passive film simultaneously reduces the solution supersaturation, thereby enabling more thermodynamically favorable and ordered growth of the corrosion product structures.

### 2.2 Microstructure Evolution of Hydroxide Films

**FIGURE 2:**
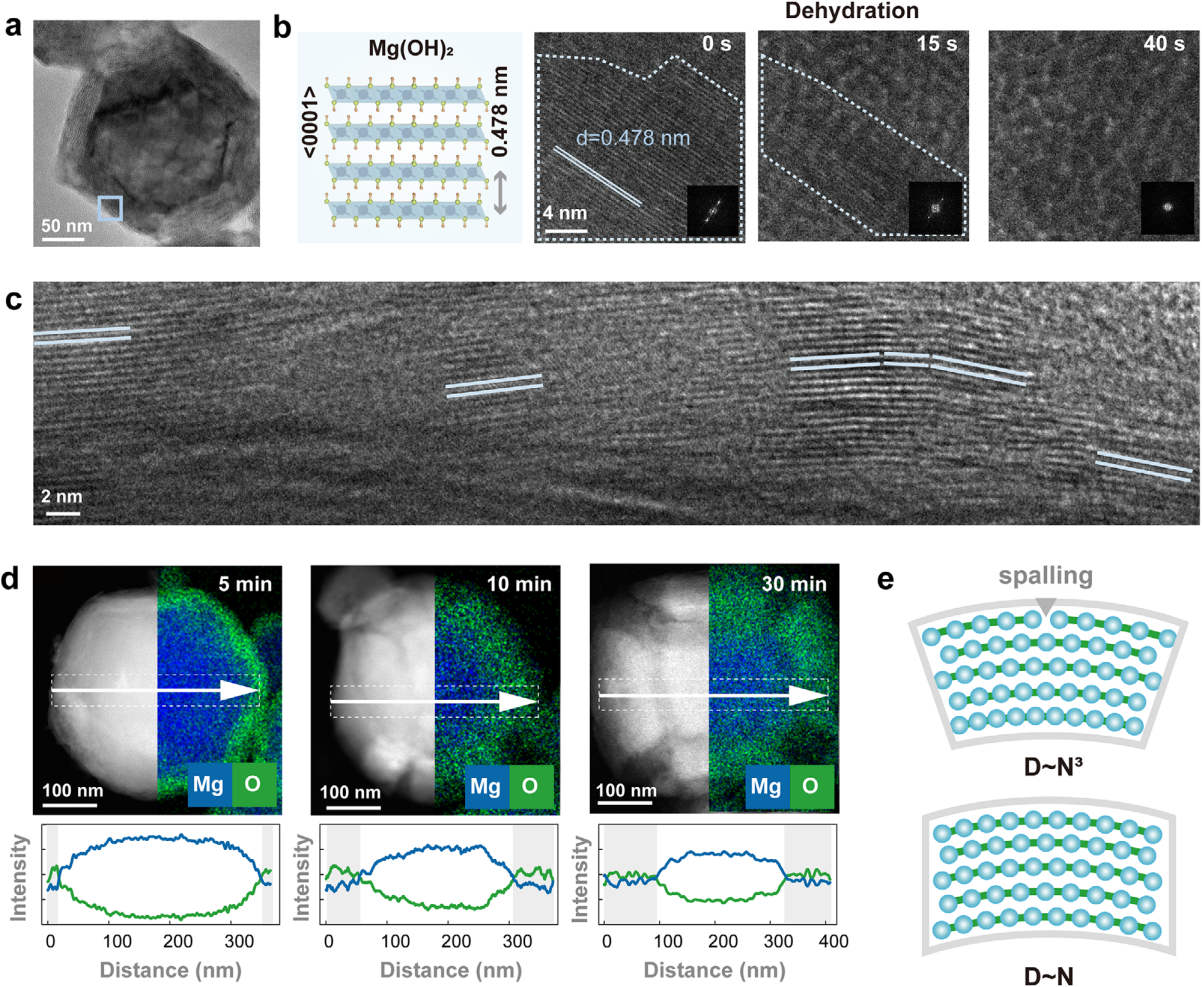
(a) TEM image of Mg nanoparticles after immersion in 1 m KOH for 30 min. (b) HRTEM image showing dehydration‐induced structural evolution within the film; a structural model of Mg(OH)_2_ is provided as an inset at the left. (c) HRTEM image of the film prior to dehydration, in which the angular deviations in lattice fringes are highlighted. (d) HAADF‐STEM images, corresponding EDS line profiles, elemental maps, and schematic diagrams of nanoparticles immersed in 1 m KOH for 5, 10, and 30 min. (e) Schematic diagrams illustrating the microstructural deformation upon bending of multilayer 2D materials with perfectly glued (top) and ultralubricated (bottom) interfaces.

While nucleation tuning theory effectively explains the more regular morphology of films formed in alkaline environments, a comprehensive understanding of magnesium's limited passivation under these conditions requires detailed insights at the atomic scale on the passive film's structure. Given that hydroxides are well‐documented to be electron beam‐sensitive and undergo dehydration under irradiation [33], we strategically select particles with extended immersion times to ensure the formation of stable films with controlled thickness (Figure 2a). We employ low‐dose electron microscopy to minimize beam damage and successfully capture the structural evolution of the film (Figure 2b). Initial high‐resolution images reveal well‐defined (0001) lattice fringes (d‐spacing = 0.47 nm) of magnesium hydroxide, which progressively disappear within 40 s of beam‐induced dehydration. The entire evolution is available in Video S1 and Figure S3. Regions with thinner films exhibit dislocation defects, which are manifested as distinct lattice misalignment and meandering (Figure 2c), providing clear evidence that crystalline perfection is intrinsically thickness‐dependent.

The formation of nanoscale ruptures at particle edges also exhibits a strong thickness dependence, as illustrated in Figure 2d for particles of comparable sizes with varying immersion duration. EDS elemental mapping and line‐scan analyses demonstrate the progressive thickening of the film from 10 to 100 nm. The corresponding low‐magnification TEM images reveal that nanoscale spalled areas at the edges form at a critical thickness of 10–20 nm and progressively widen with increasing film thickness. Because the mechanical properties of materials at the nanoscale are intrinsically governed by their crystal structure and bonding characteristics, the formation of nanoscale rupture in magnesium hydroxide films reflects a fundamental relationship between film thickness and mechanical stability, specifically, the scaling of bending rigidity (D) with layer number (N), as described for multilayer 2D materials. As illustrated in the schematic diagram in Figure 2e, classical plate theory predicts that D is proportional to N^3^ for perfectly bonded multilayers [34]. In contrast, for typical van der Waals materials (e.g., graphene) with weak interlayer interactions, the ultralubricated interfaces promote independent bending of each layer. The weak interaction thus causes the overall bending rigidity to scale linearly with the number of layers (D ∝ N) [35, 36]. The hydrogen‐bonded interlayer interactions in magnesium hydroxide give rise to an intermediate bonding strength, which lies between fully bonded bulk materials and completely lubricated van der Waals systems. This intermediate bonding state thus provides intermediate coupling strength. Consequently, when the film reaches a critical thickness of 10–20 nm, the accumulated strain energy must be released through spalling; as the film thickness further increases, subsequent spall propagation occurs.

### 2.3 Epitaxial Mg(OH)_2_/Mg Interface with Atomically Flat Morphology

**FIGURE 3:**
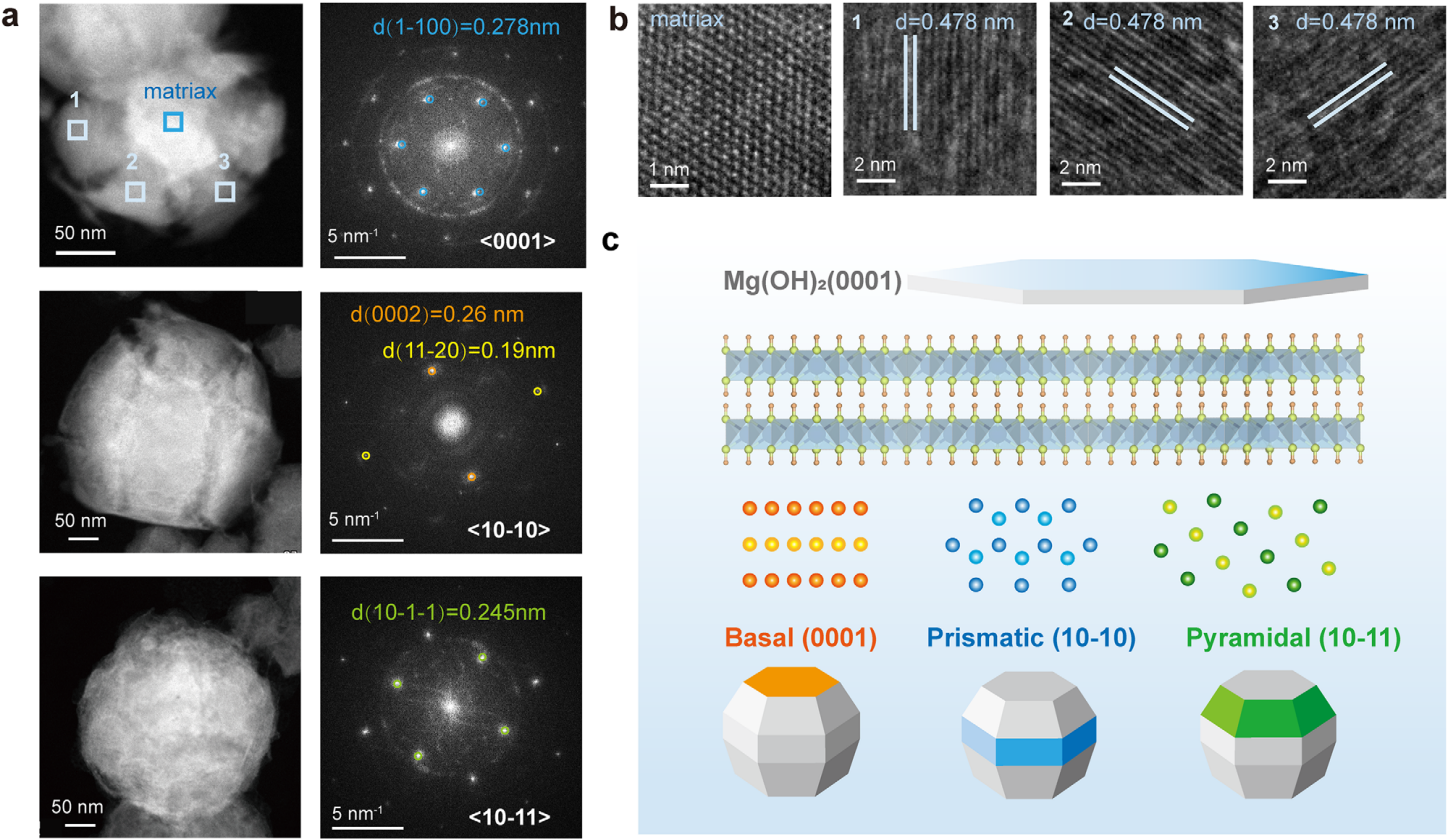
(a) TEM image of nanoparticles oriented along the <0001>, <10‐10>, and <10‐11> zone axes. after 30 min of immersion in 1 m KOH. (b) HRTEM image of the matrix region (dark blue area in (a)) and the film region (light blue area in (a)). (c) Schematic model of lattice‐matched nucleation at the Mg(OH)_2_/Mg interface.

Building upon the understanding of intrinsic spalling within the film, we now examine the film/substrate interface that plays a key role in the initial passivation behavior. Although electron beam irradiation‐induced damage presents a challenge as it prevents pristine atomic‐scale imaging of the intact interface, the nanoscale structural and morphological correspondence between the metallic substrate and hydroxide film still provides a robust characterization criterion. We orient the nanoparticles along specific crystallographic directions to image the cross‐section of the interface. Figure 3a and Figure S4 show TEM images of nanoparticles oriented along the <0001>, <10‐10>, and <10‐11> zone axes after 30 min of immersion in 1 m KOH. It can be observed that the growth mode of magnesium hydroxide on the three characteristic planes of magnesium is consistently lateral and strictly parallel to the substrate, with no evidence of vertical growth or layers perpendicular to the substrate. This is also clearly demonstrated in the HRTEM in Figure 3b, where the lattice arrangement of the film corresponds to the lamellar structure.

This growth behavior differs distinctly from that of the oxide films. In typical gas‐phase oxidation, the process of oxide formation involves a solid–solid phase transformation where the film and substrate maintain a strict epitaxial relationship [37]. When transitioning to a more humid environment, in situ oxidized aluminum film formed in water vapor exhibits a similar yet less stringent epitaxial relationship, considered a product of structural reorganization during the process [38]. In contrast, under fully liquid‐phase conditions during aqueous oxidation, growth is dominated by the complex evolution of the liquid–solid interface and is primarily controlled by supersaturation (S), analogous to the growth pattern of electrodeposited films [39, 40]. The environmental driving forces across gas, vapor, and liquid phases govern the intermediate growth processes, which ultimately dictate the resulting interfacial structure and properties of corrosion film.

Figure 3c schematically illustrates the interfacial relationships within laterally grown magnesium hydroxide films, highlighting significant misfit variations across the three distinct interfaces. The Mg substrate has a hexagonal close‐packed (HCP) structure, while the magnesium hydroxide film exhibits a hexagonal layered structure. Specifically, the Mg_(0001)_/Mg(OH)_2(0001)_ interface demonstrates high lattice matching, with in‐plane lattice parameters of a_(Mg)_ = 0.320 nm and a_(Mg(OH)2)_ = 0.314 nm. The interfacial misfit (δ) was calculated based on δ = |a_2_ − a_1_| / [(a_2_ + a_1_)/2], and is approximately 1.9%, well below the 5% threshold, promoting the formation of a coherent interface. This atomic registry lowers the interfacial energy and facilitates 2D lateral epitaxial growth, fostering a compact and adherent interfacial layer that acts as an effective barrier against corrosive species. In contrast, the lateral interfaces exhibit significantly larger misfits due to anisotropic lattice expansion. For the prismatic (10‐10) interface, the misfit along the c‐axis reaches ∼4.3%; for the pyramidal (10‐11) interface, the geometric misfit exceeds ∼8.1%. These high‐misfit interfaces (approaching or exceeding 5%) are characterized by high interfacial energy and poor adhesion, serving as preferential pathways for corrosion propagation.

### 2.4 Mesoscale Visualization of Chloride‐Driven Corrosion Propagation

**FIGURE 4:**
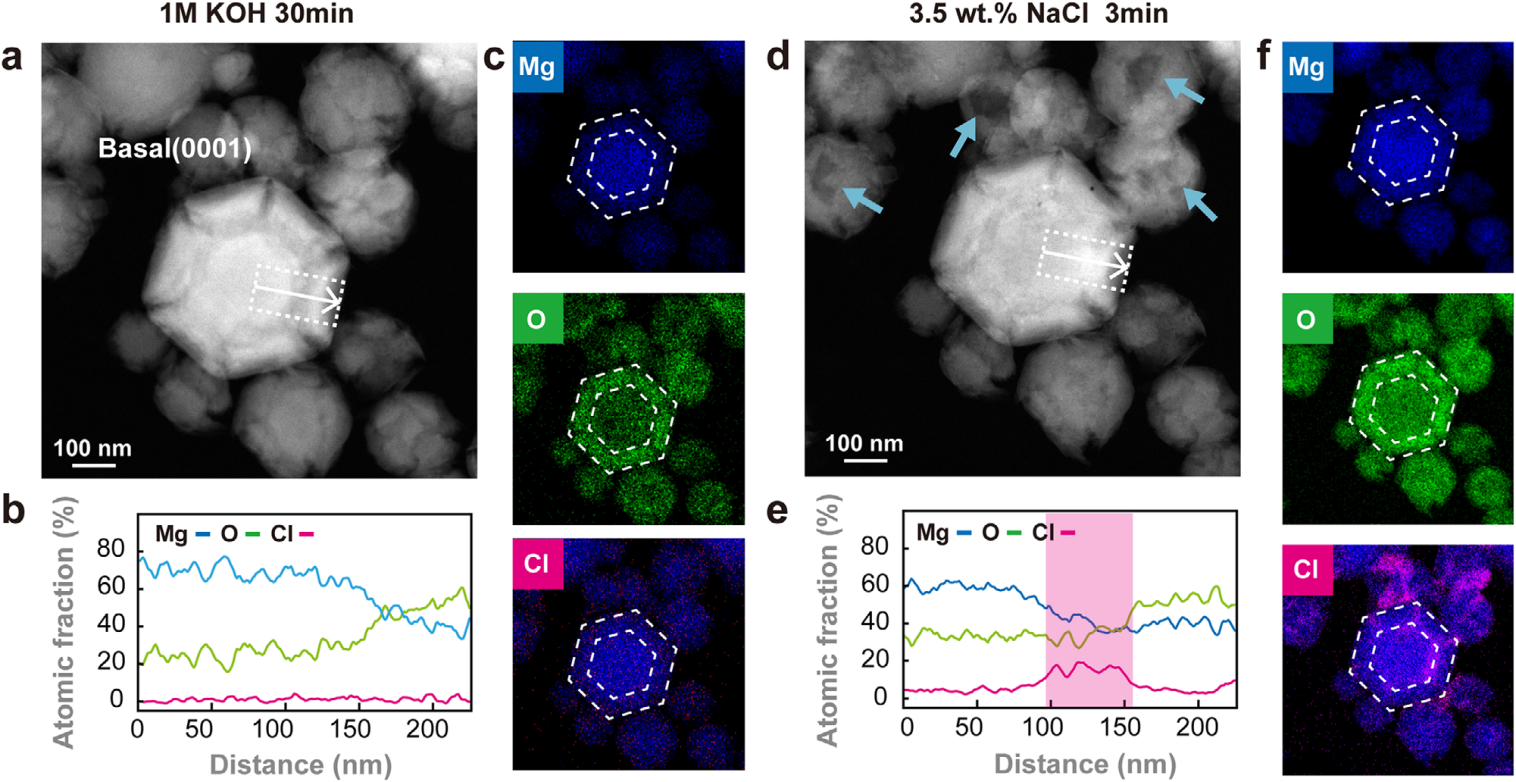
Quasi‐in situ characterization of the same nanoparticle after (a–c) immersion in 1 m KOH for 30 min and (d–f) subsequent exposure to 3.5 wt.% NaCl for 3 min. (a, d) HAADF‐STEM images, blue arrows indicate that some small particles have been rapidly corroded and hollowed out; (b, e) EDS line profiles acquired along the white dashed arrows in (a, d), respectively; (c, f) EDS elemental maps.

To directly test the corrosion and chloride etching resistance of the film studied above, we expose the passivated single‐crystalline Mg nanocrystals to a chloride‐containing corrosive medium and image the treated samples. This approach offers direct visualization of site‐specific attack propagation, tracing how nanoscale structural features govern macroscopic degradation. Figure 4 presents quasi‐in situ characterization of the same Mg nanoparticle, revealing a direct spatial correlation between pre‐existing spalled areas and chloride etching. After 30 min of initial immersion in 1 m KOH (a–c), in the HAADF‐STEM image (Figure 4a), we identify features with surface nanoscale ruptures. After subsequent exposure to 3.5 wt.% NaCl for just 3 min, Figure 4d–f show that the chloride ions preferentially penetrate through these nanoscale ruptures. The EDS elemental mapping (Figure 4f; Figures S5 and S6) displays concentrated Cl^−^ accumulation at spalled areas, while EDS line profiles (Figure 4e) confirm the chlorine penetration depth aligned with spall propagation trajectories rather than uniform surface adsorption. This nanoscale observation provides direct evidence that pre‐existing structural defects, particularly ruptures formed during initial hydroxide growth, serve as primary entry pathways for corrosive chloride species. Importantly, such nano‐film ruptures are present not only in nanoscale particles, but also in macroscopic passive films. Film ruptures at the nanoscale can often escape conventional detection techniques, but their existence can compromise the protection integrity. These hidden chloride pathways can cause catastrophic localized corrosion.

**FIGURE 5:**
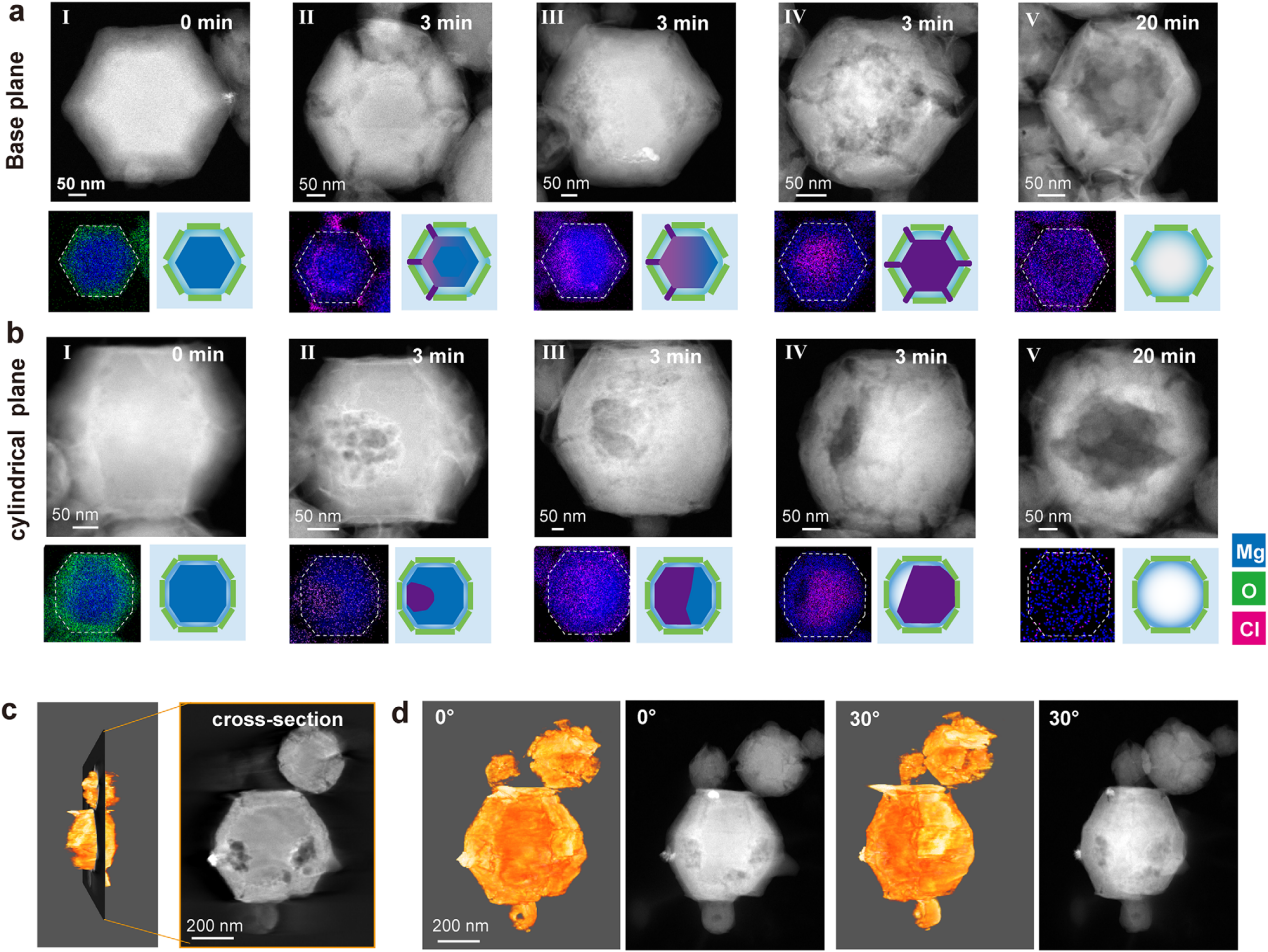
Corrosion evolution of nanoparticles in 3.5 wt.% NaCl solution observed by HAADF‐STEM and EDS mapping. (a) View along the basal plane and (b) view along the prismatic surface, each showing the initial state (0 min), intermediate stage (3 min), and final state (20 min) of chloride‐induced corrosion, along with their corresponding elemental distributions. (c) 3D reconstruction (left) and central cross‐sectional slice (right) of the corroded nanoparticle. (d) Reconstructed particle and corresponding HAADF‐STEM images viewed at 0° and 30° tilt angles.

With analysis of numerous particles at different corrosion stages, we can construct a chronological sequence to further clarify the chloride invasion and evolution pathways. By imaging the nanoparticles along two orientations, perpendicular and parallel to the (0001) basal plane, we obtain comprehensive structure and composition information across the corrosion states. The initial images in Figure 5a,b show particles covered with an intact film, which are corrosion‐free. Images II–IV depict three distinct corrosion stages after immersion: mildly etched, moderately etched, and fully etched. As the dense Mg structure transforms into loose corroded products, the HAADF‐STEM image contrast decreased significantly. Along the ⟨0001⟩ basal plane direction, Figure 5a and Figure S7 show no clear contrast variation or corrosion front, suggesting minimal or uniform etching in this orientation. In comparison, imaging along the prismatic surface direction (Figure 5b; Figure S8) reveals a sharp interface between the metallic core and corrosion products, indicating preferential etching along the prismatic planes. Image V shows the final state after 20 min of immersion: chloride ions are entirely removed, the Mg core is completely hollow, and the hydroxide shell remains structurally intact. Without chloride interference, etching traces on the prismatic surface are more distinct, underscoring the role of crystallographic orientation in corrosion evolution (Figure S9).

To understand the progression of Mg corrosion in 3D, we perform electron tomography of the Mg nanoparticles after 20 min of immersion. Figure 5c displays a tomographic reconstruction and a central cross‐sectional slice of the nanoparticle, visually confirming that etching and chloride invasion begin at the spalled areas, consistent with earlier findings, and progress inward from the film‐metal interface. Contrary to the traditional model of uniform dissolution, the 3D imaging shows that the exterior hydroxide shell remains intact with a plate‐like morphology, while significant corrosion occurs inside the metallic core. Figure 5d presents reconstructed particle views and corresponding HAADF‐STEM images at tilt angles of 0° and 30°, highlighting the morphological details of the corrosion front and retained shell across orientations (see Video S2 for a full 3D rotation). Together, these results demonstrate the spatially heterogeneous nature of corrosion attack and the critical role of nanoscale ruptures in sub‐surface chloride penetration.

The observed anisotropic etching is not incidental but originates from the inherent differences of the interface between the film and different Mg surface planes. As shown in Figure 3c, interfaces between the film and surface planes along the prismatic direction exhibit higher lattice mismatch compared to that with the Mg basal plane. After chloride infiltration through nanoscale ruptures, corrosion preferentially begins at these interfaces with high lattice mismatch, exposing the unprotected substrate and creating channels for further chloride penetration into the metal. Without protection from the passive film, etching proceeds along the non‐close‐packed prismatic direction, due to weak crystallographic bonding.

Based on our systematic investigation, we have elucidated the multiscale formation and spalling mechanisms of the magnesium hydroxide passivation film on Mg nanoparticles, spanning from atomic‐scale lattice matching to microscale film growth and rupture. These findings provide a mechanistic clarification of corrosion initiation, helping to resolve long‐standing controversies regarding magnesium's passivation behavior. Bridging our mechanistic understanding with material design, we propose that a hybrid barrier architecture featuring an atomically coherent interface in combination with a spall‐resistant ductile overlayer can synergistically improve corrosion resistance. For instance, improving interfacial coherence through lattice‐matching can strengthen the initial adhesion, while incorporating lubricating interlayers such as 2D materials or organic species can effectively dissipate the strain and suppress spall propagation. Furthermore, forming amorphous or nanocomposite surface films via alloying or surface treatment can also be a promising route toward improved film compactness and uniformity. Collectively, these insights open new avenues for designing next‐generation corrosion‐resistant Mg alloys with tailored interfacial and mechanical properties.

## 3 Conclusions

In summary, by employing single‐crystalline nanocrystals as model systems for direct nanoscale observation, we provide a mechanistic clarification of corrosion initiation. Specifically, we demonstrate that the protective capacity of Mg(OH)_2_ passive films on magnesium is governed by their nanoscale integrity and interfacial coherence. These nanoscale defects and lattice mismatches remain concealed beneath a macroscopically intact passive film, yet drive unpredictable local corrosion of the underlying magnesium substrate. In alkaline environments, a dense surface film initially forms, but as it thickens, accumulated strain energy from bending rigidity and substrate undulation causes nanoscale spalling. These film ruptures allow chloride ions to penetrate and preferentially attack the Mg(OH)_2_/Mg interface rather than dissolving the bulk film, resulting in localized substrate corrosion. The degradation severity is strongly influenced by lattice mismatch of the Mg(OH)_2_/Mg interface: prismatic {10‐10} and pyramidal {10‐11} planes undergo more severe corrosion than the basal (0001) plane, which supports better epitaxial matching. Our findings illuminate the previous discrepancies between thermodynamic predictions and experimental observations and provide key insights for designing corrosion‐resistant, lightweight magnesium‐based materials.

## 4 Experimental Section/Methods

### 4.1 Sample Preparation

Single‐crystal magnesium nanoparticles were produced by a direct current (DC) arc plasma evaporation method. The process was conducted using an apparatus comprising a reaction chamber and a collecting room. Bulk pure Mg with a purity of 99.9% was used as the anode material for evaporation. To ensure safety and prevent oxidation upon exposure to air, the resultant ultrafine Mg particles were slowly passivated within the chamber using a mixture of argon and air before being collected for subsequent analysis.

### 4.2 Characterization

The nanoparticles were first subjected to immersion corrosion in 1 m KOH solution and in deionized water to facilitate the growth of a corrosion product film on their surfaces. Subsequently, nanoparticles with the surface film underwent a further immersion corrosion process in a medium of 3.5 wt.% NaCl solution. This two‐step protocol is designed to first form a protective or modified layer on the nanoparticles in an alkaline environment, followed by an assessment of the film's stability or further transformation in a saline solution, which is a common corrosive medium. Quasi in situ corrosion characterization was performed on individual particles deposited on TEM grids. The identical locations of the particles were first imaged using TEM prior to corrosion. Subsequently, the grids were subjected to the corrosive medium. Following the corrosion exposure, the same locations were re‐imaged to track the structural and morphological evolution.

Transmission Electron Microscopy (TEM) imaging, 3D reconstruction analyses, and Energy dispersive x‐ray Spectroscopy (EDS) analyses were conducted in a Talos F200X G2 microscope operated at an accelerating voltage of 200 kV. To mitigate electron beam effects, low‐dose imaging conditions (beam current ∼0.5 nA; estimated dose rate ∼3.1 × 10^3^ e^−^ Å^−^^2^ s^−^^1^) were strictly applied. This minimized beam‐induced artifacts and enabled the accurate capture of structural evolution.

To ensure the statistical significance and reliability of the observations, the experiment was repeated multiple times, and a sufficiently large number of particles were characterized to confirm that the observed corrosion phenomena were reproducible and not attributable to artifacts related to sample preparation.

## Funding

National Natural Science Foundation of China (Nos. 52271008, 52525106, and 52127801).

## Conflicts of Interest

The authors declare no conflicts of interest.

## Supporting information




**Supporting File 1**: advs75169‐sup‐0001‐SuppMat.docx.

**Supporting File 2**: advs75169‐sup‐0002‐VideoS1.mp4.

**Supporting File 3**: advs75169‐sup‐0003‐VideoS2.avi.

## Data Availability

The data that support the findings of this study are available from the corresponding author upon reasonable request.
