# Supplementary material for: Site‐Specific Hydroxide Formation and Corrosion on Mg Nanocrystals
Source: Adv Sci (Weinh). 2026 Apr 27;13(36):e75169. doi: 10.1002/advs.75169 (PMC13317796; doi:10.1002/advs.75169)
Supplement: Supplementary file 1 — Supporting File 1: advs75169‐sup‐0001‐SuppMat.docx. [file ADVS-13-e75169-s002.docx]

**Supporting Information**

**Site-Specific Hydroxide Formation and Corrosion on Mg Nanocrystals**

Yao Liu^1,3^, Zongmin Sun^1,3^, Chenhao Wu^2,3,4^, Kun Yang^3,4^, Zhiyuan Ge^3,4^, Chenyu Wang^1,3^, Wenpei Gao^3,4^*, Jianbo Wu^2,3,4^*, Xiaoqin Zeng^1,3^*.

^1^ National Engineering Research Center of Light Alloy Net, Shanghai Jiao Tong University, Shanghai, PR China.

^2^ Center of Hydrogen Science, Shanghai Jiao Tong University, 800, Shanghai, PR China.

^3^ State Key Laboratory of Metal Matrix Composites, School of Materials Science and Engineering, Shanghai Jiao Tong University, 800, Shanghai, PR China.

^4^ Future Material Innovation Center, Zhangjiang Institute for Advanced Study, Shanghai Jiao Tong University, Shanghai, PR China.

* Corresponding author: [gaowenpei@sjtu.edu.cn](mailto:gaowenpei@sjtu.edu.cn), [jianbowu@sjtu.edu.cn](mailto:jianbowu@sjtu.edu.cn), [xqzeng@sjtu.edu.cn](mailto:xqzeng@sjtu.edu.cn).


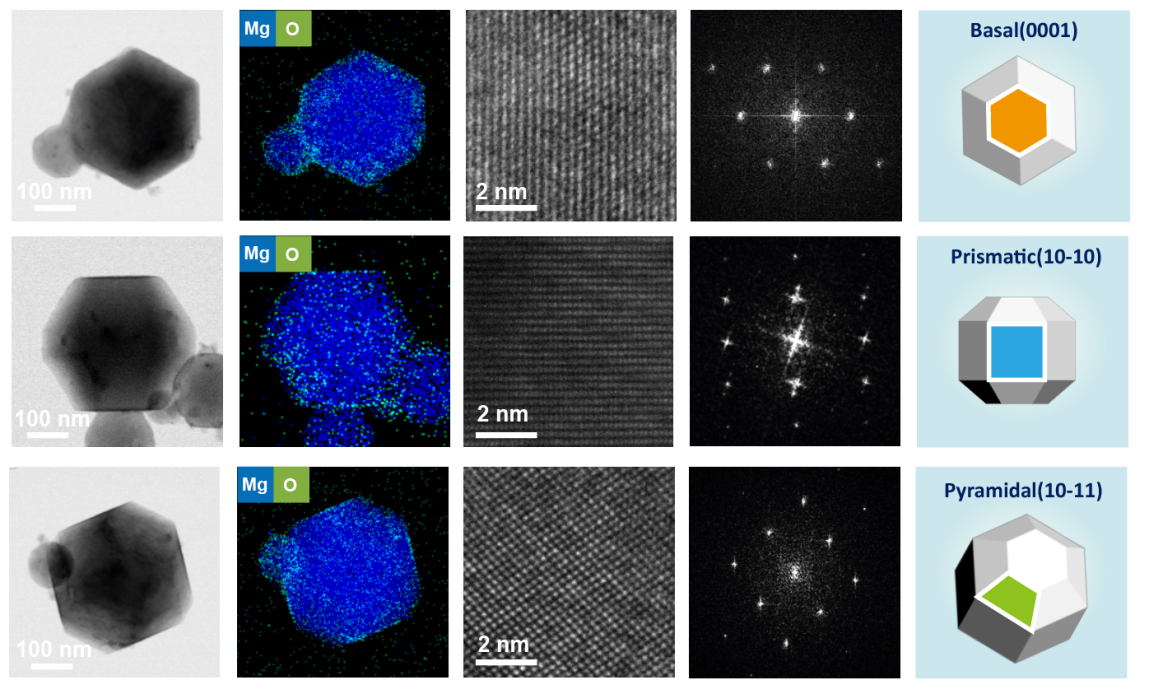


**Figure S1. TEM image EDS mapping of the Mg nanoparticles viewed by <0001>, <10-10>, <10-10>.** Additional structural analysis of the faceted nanoparticles along different zone axes. This figure supplements the analysis presented in Figure 1 by showing high-resolution TEM images and corresponding FFT of nanoparticles oriented along other crystallographic directions, further confirming the identified crystal planes and overall symmetry.


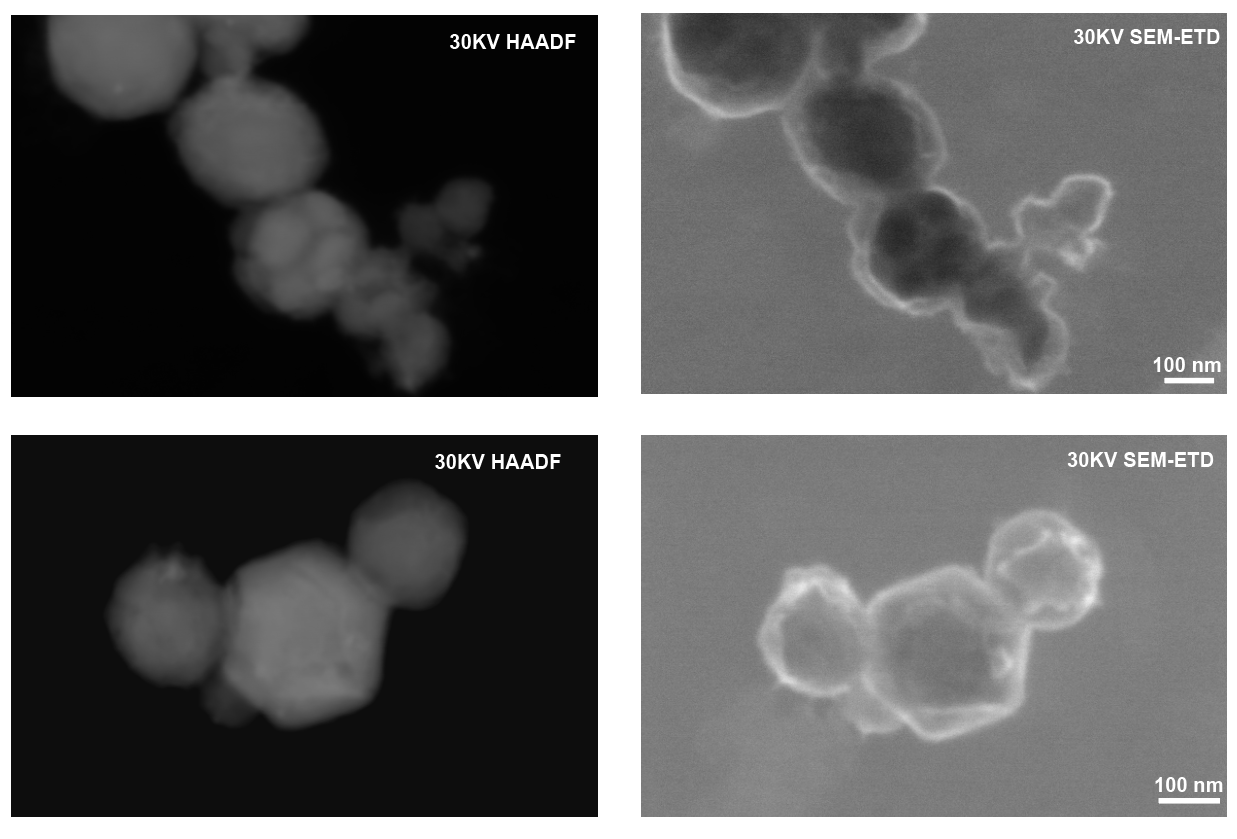


**Figure S2. SEM image of the Mg nanoparticles after being immersed in 1 M KOH for 5 minutes.** Leveraging its superior depth of field for three-dimensional surface visualization, clearly resolves the three-dimensional plate-like structures were formed, and these structures exhibited site-specific growth patterns: continuous coverage was observed on planar facets, while distinct nanoscale spalled areas were present at edges and corners.


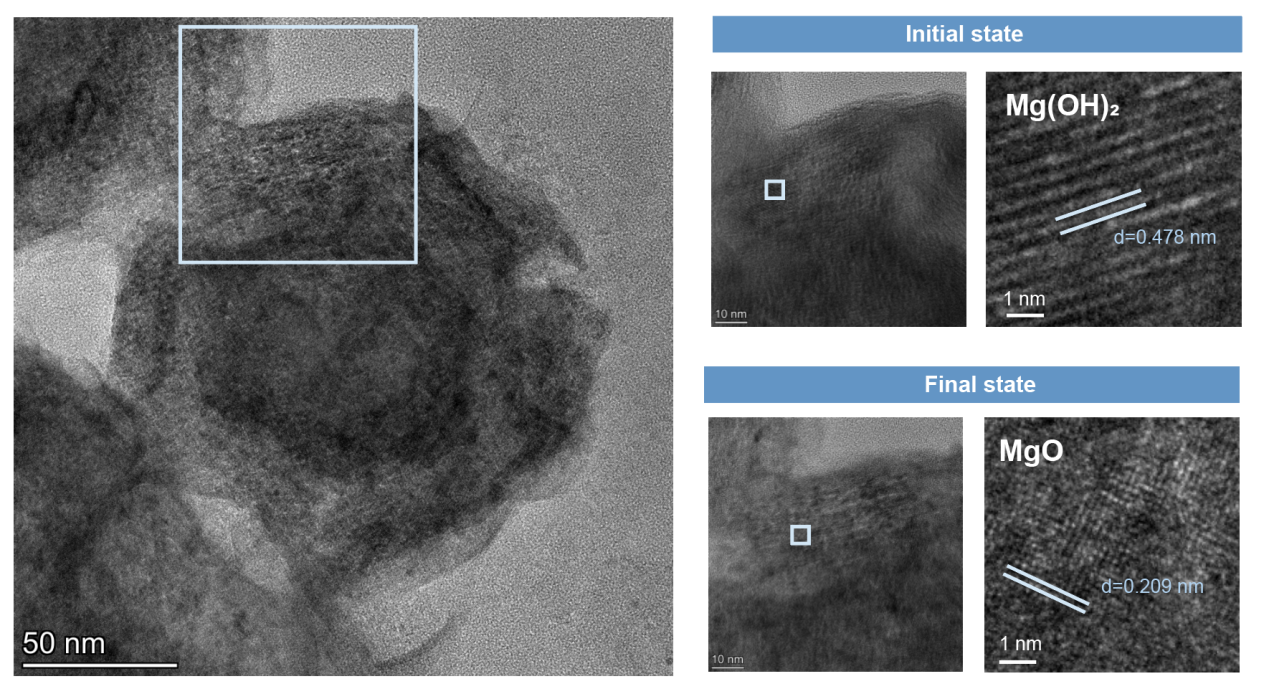


**Figure S3. HRTEM image showing the dehydration of the film from magnesium hydroxide to magnesium oxide.** The initial state is viewed along the [0001] zone axis of Mg(OH)₂, evidenced by the characteristic lattice fringes. The final state confirms the complete dehydration of the film, as indicated by the transformation to the [001] zone axis of MgO.


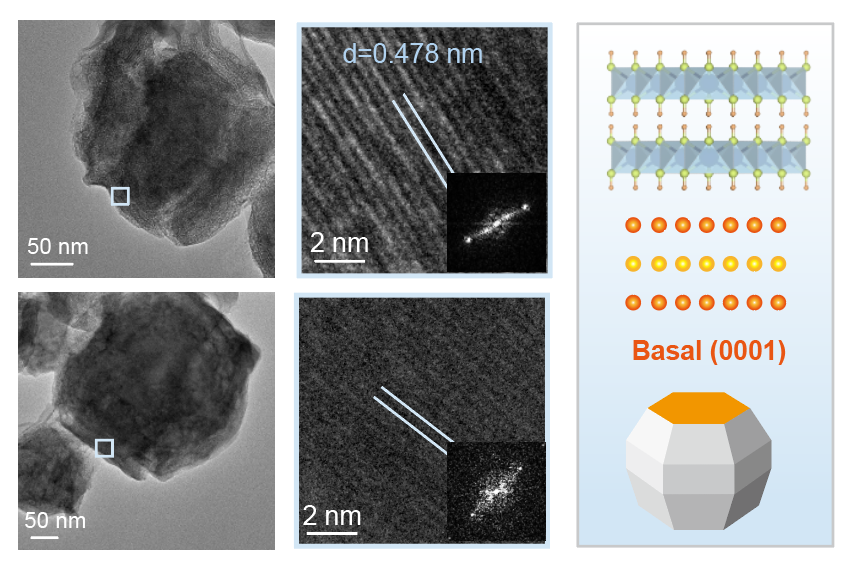


**Figure S4. TEM image of film morphology on nanoparticle oriented along <10-10> after being immersed in 1 M KOH for 30 minutes.** Additional HRTEM evidence supporting the lateral growth mode of Mg(OH)₂. Images of nanoparticles oriented along different zone axes further confirm that the growth of the hydroxide film occurs parallel to the Mg substrate, with no observed vertical growth component.


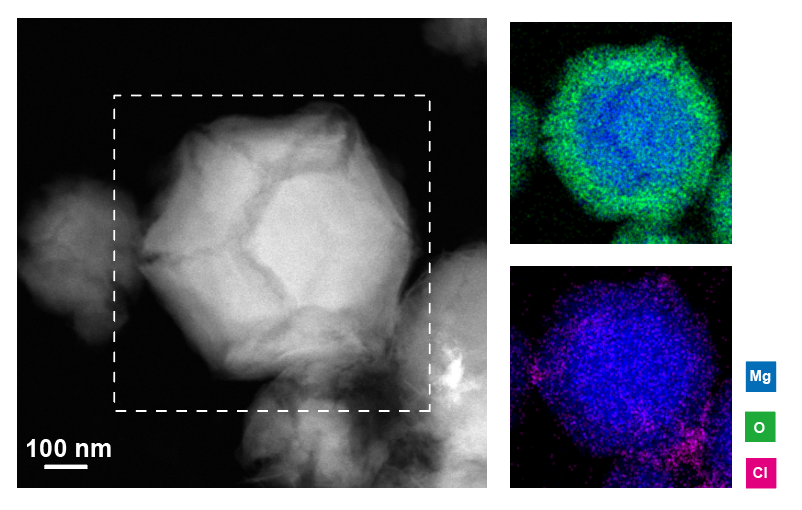


**Figure S5. Side view of the Cl^-^ attack initiation on nanoparticle.** This image provides a complementary perspective to Figure 4. The side-view angle corroborates that Cl⁻ penetration follows the trajectory of nanoscale ruptures into the structure.


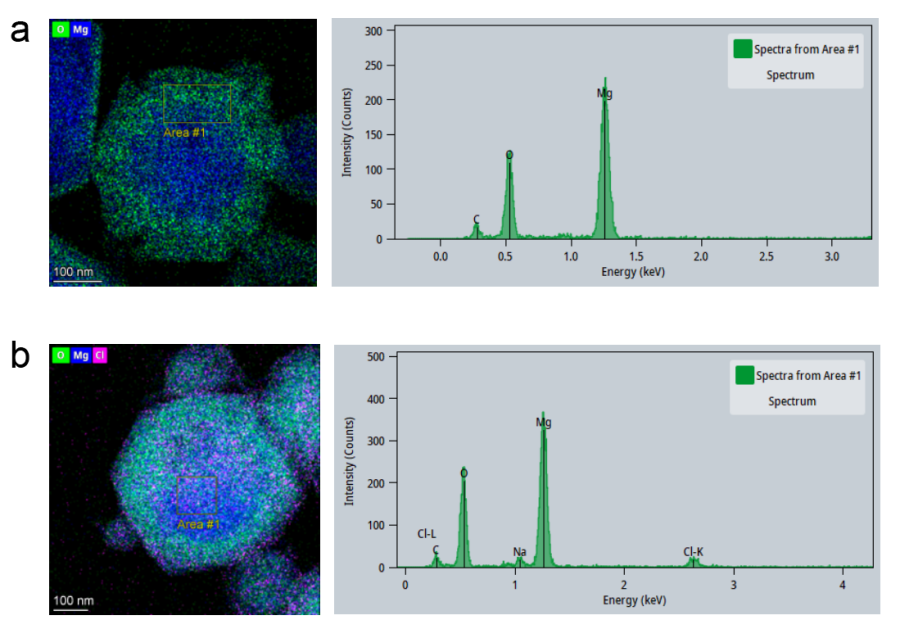


**Figure S6. EDS point spectra of nanoparticles before (a) and after (b) immersion in 3.5 wt.% NaCl solution for 20 minutes.** The spectra serve as a crucial supplement to the elemental mapping data. The definitive Cl peak detected after corrosion (b) offers qualitative confirmation of chlorine presence at the analyzed points, corroborating the mapping results.


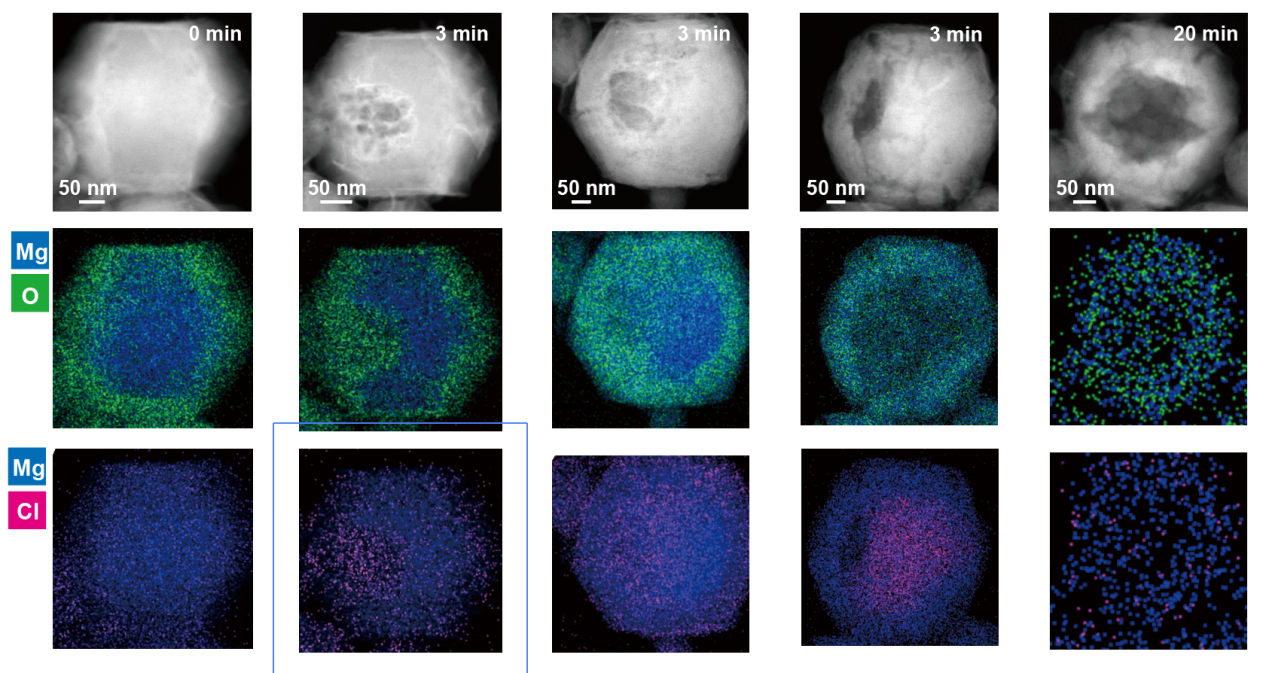


**Figure S7. Detailed EDS mapping complementing Figure 5a.** This expanded view includes the distributions of oxygen and magnesium, providing full chemical context for the chlorine infiltration and the associated corrosion products at the attack site.


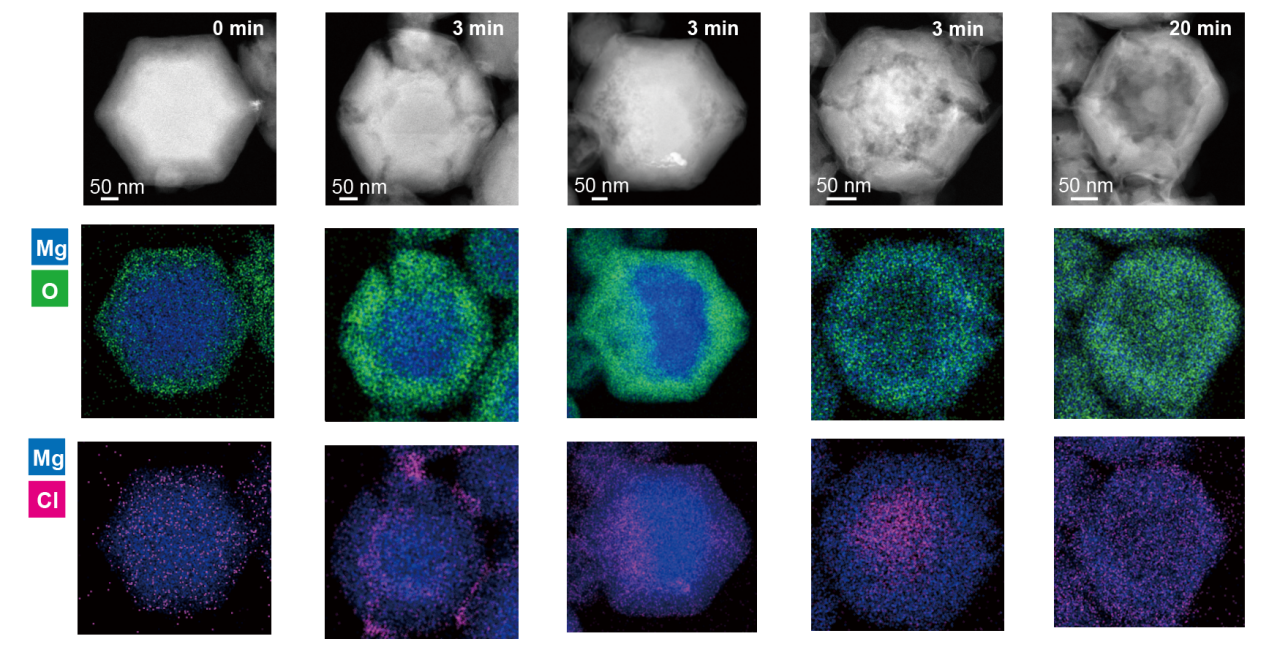


**Figure S8. Detailed EDS mapping complementing Figure 5b.** This expanded view includes the distributions of oxygen and magnesium, providing full chemical context for the chlorine infiltration and the associated corrosion products at the attack site.


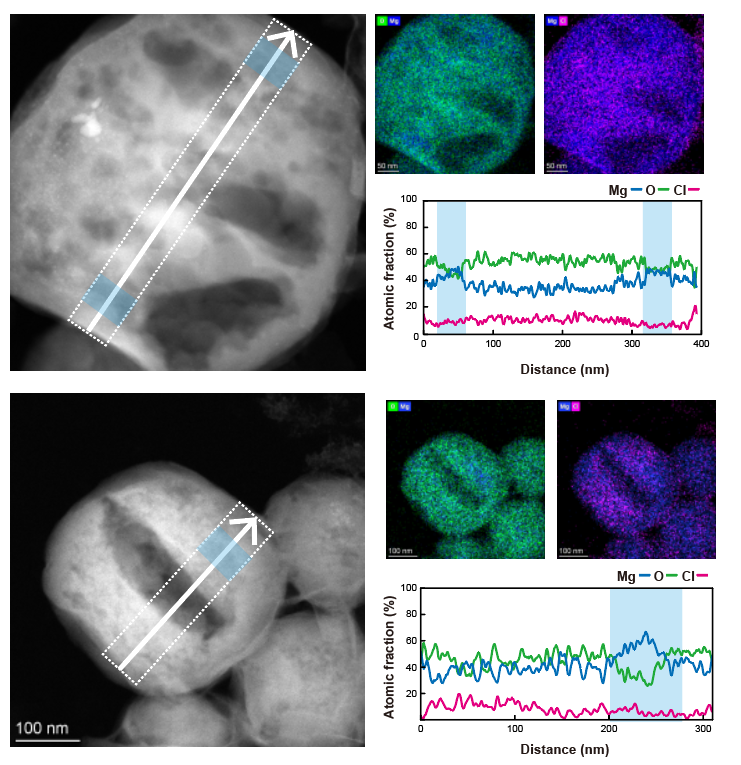


**Figure S9. EDS elemental mapping and line profiles of nanoparticles after immersion in 3.5 wt.% NaCl solution for 20 minutes.** Two nanoparticles exhibiting similar anisotropic corrosion morphology were analyzed. Both reveal through-thickness corrosion traces perpendicular to the (0001) plane. Their corresponding EDS line profiles demonstrate the presence of an unetched magnesium metal core along the [0001] direction. This provides direct evidence that the corrosion process is hindered along the c-axis, highlighting the crystallography-dependent nature of the attack.
